# Two Lineages of Dengue Virus Type 2, Brazil

**DOI:** 10.3201/eid1603.090996

**Published:** 2010-03

**Authors:** Michelli Faria Oliveira, Josélio Maria Galvão Araújo, Orlando Costa Ferreira, Davis Fernandes Ferreira, Dirce Bonfim Lima, Flavia Barreto Santos, Hermann Gonçalves Schatzmayr, Amilcar Tanuri, Rita Maria Ribeiro Nogueira

**Affiliations:** Universidade Federal do Rio de Janeiro, Rio de Janeiro, Brazil (M.F. Oliveira, O. Costa Ferreira Jr., D.F. Feirreira, A. Tanuri); Instituto Oswaldo, Rio de Janeiro (J.M.G. Araújo, F.B. Santos, H.G. Schatzmayr, R.M. Ribeiro Nogueira); Universidade do Estado do Rio de Janeiro, Rio de Janeiro (D.B. Lima); 1 These authors contributed equally to this article.

**Keywords:** Dengue viruses, DENV-2, vector-borne, infections, outbreak, viruses, dengue, Brazil, letter

**To the Editor:** Dengue viruses (DENVs) belong to the genus *Flavivirus* (family *Flaviviridae*) and exist as 4 antigenic types, serotypes 1–4, each with well-defined genotypes. Dengue virus is associated with clinical manifestations that range from asymptomatic infections and relatively mild disease (classic dengue fever) to more severe forms of dengue hemorrhagic fever and dengue shock syndrome. Dengue has become one of the most serious vector-borne diseases in humans. The World Health Organization estimates that 2.5 billion persons live in dengue-endemic areas and >50 million are infected annually (*1*).

In 1986, dengue virus type 1 (DENV-1) caused an outbreak in the state of Rio de Janeiro and has since become a public health concern and threat in Brazil. (*2*). In 1990, DENV-2 was reported in the state of Rio de Janeiro, where the first severe forms of dengue hemorrhagic fever and fatal cases of dengue shock syndrome were documented. The disease gradually spread to other regions of the country (*3*). In 2002, DENV-3 caused the most severe dengue outbreak in the country and sporadic outbreaks continued to be documented through 2005 (*4*).

Since 1990, two additional epidemics caused by DENV-2 have occurred (1998 and 2007–2008) in Brazil. A severe DENV-2 epidemic in the state of Rio de Janeiro began in 2007 and continued in 2008; a total of 255,818 cases and 252 deaths were reported (*5*). This epidemic prompted us to investigate the genetic relatedness of DENV-2 for all of these epidemics.

DENV-2 isolates from these epidemic periods were subjected to sequencing and comparison. Gross sequences of DENV-2 isolates from all epidemic periods grouped with sequences from DENV-2 American/Asian genotype; this finding was expected because this genotype is circulating in the Americas (*6**,**7*). Sequences of DENV-2 isolates from the 1998 epidemic grouped with sequences of DENV-2 isolates from the 1990 epidemic (data not shown) suggesting that viruses circulating during these 2 epidemic periods belong to the same lineage of the DENV-2 strain originally found in the state of Rio de Janeiro. However, sequences of DENV-2 isolates from 2007/2008 epidemics grouped separately and distinctly from the 1990 and 1998 DENV-2 isolates and represented a monophyletic group in the phylogenetic tree with bootstraps of 98% (Figure). This result shows a temporal circulation of genetically different viruses in Rio de Janeiro that could be a result of local evolution of DENV-2 since its introduction in 1990, or even an introduction of a new lineage of DENV-2 in the region.

**Figure:**
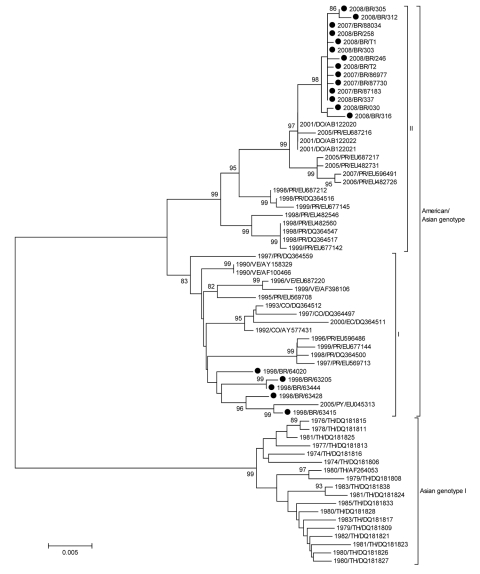
Neighbor-joining phylogenetic tree of 68 complete envelope (E) gene sequences of dengue virus type 2 (DENV-2). Only bootstrap values >80% are shown. DENV-2 sequences obtained from 21 patients infected during the 1990, 1998, and 2007–2008 epidemics were isolated from acute-phase cases. Viral RNA was extracted from 140 µL of serum or supernatant of infected C636 cell cultures by using the QIAamp Viral RNA Mini Kit (QIAGEN, Valencia, CA, USA), and cDNA synthesis was performed by using the ThermoScript reverse transcription–PCR System (Invitrogen, Carlsbad, CA, USA) according to the manufacturers’ recommendations. We obtained a fragment of 1,878 bp with the complete dengue virus envelope (E) gene sequence by seminested PCR using Taq Platinum DNA polymerase (Invitrogen). The PCR products were purified by using the Millipore (Billerica, MA, USA) purification column and sequenced by using BigDye Terminator version 3.1 Cycle Sequencing (Applied Biosystems, Foster City, CA, USA) and ABI3130 automated sequencer. Sequences of the E gene were compared with DENV-2 sequences of American/Asian genotype deposited in GenBank (www.ncbi.nlm.nih.gov). Strains of Asian genotype I served as the outgroup. All sequences were aligned by using ClustalX (www.ebi.ac.uk/clustalw), and phylogenetic analysis was performed by using MEGA 4.0 (www.megasoftware.net), according to the Tamura-Nei model. First, neighbor-joining trees were constructed by using a 798 pb of the at 5′ region of the E gene and later trees were constructed with sequences from the 1998, and 2007–2008 epidemics by using the complete E gene sequence (1,485 bp). The reliability of the inferred phylogenetic tree was estimated by the bootstrap method, with 1,000 replications. Horizontal branch lengths are drawn to scale, and the tree was rooted by using the Asian genotype, which always appears as the most divergent. Scale bar represents percentage of genetic distance. The names of DENV-2 isolates include reference to year of isolation and country of origin: BR, Brazil; CO, Colombia; DO, Dominican Republic; EC, Ecuador; PR, Puerto Rico; PY, Paraguay; TH, Thailand; VE, Venezuela. GenBank accession nos: 1990/BR/39537 (GQ368156); 1990/BR/40044 (GQ368157); 1998/BR/63415 (GQ368158); 1998/BR/63428 (GQ368159); 1998/BR/63205 (GQ368160); 2007/BR/86977 (GQ368161); 2007/BR/87183 (GQ368162); 2007/BR/87730 (GQ368163); 2007/BR/88034 (GQ368164); 2008/BR/030 (GQ368165); 2008/BR/246 (GQ368166); 2008/BR/258 (GQ368167); 2008/BR/303 (GQ368168); 2008/BR/305 (GQ368169); 2008/BR/312 (GQ368170); 2008/BR/316–08 (GQ368171); 2008/BR/337–08 (GQ368172); 1998/BR/63444 (GQ368173); 1998/BR/64020 (GQ368174); 2008/BR/T2 (GQ368175); 2008/BR/T1 (GQ368176).

A study conducted by Aquino et al. (*7*) showed that Paraguayan DENV-2 strains could be grouped as 2 distinct variants within the American/Asian genotype, thus further supporting that the introduction of new DENV-2 variants may likely associate with the shift of dominant serotypes from DENV-3 to DENV-2 in 2005 and might have caused an outbreak of DENV-2. Our results are consistent with this scenario because was a shift of a dominant serotype from DENV-3 to DENV-2 that was observed in 2008 in Rio de Janeiro. However, other factors, such as immunity level to DENV-3 and DENV-2, could explain the shift of dominant serotype besides the circulation of a new viral variant.

Because the dengue outbreaks of 2007 and 2008 were the most severe of the dengue infections in Brazil in terms of number of cases and deaths, this genetically distinct DENV-2 could have contributed to this pathogenic profile. Additionally, these samples came from disperse locations in Rio de Janeiro and we do not believe that there is a clustering issue in our sampling. However, again, other factors must be considered as contributors to this scenario because of the intrinsic properties of this distinct virus, host susceptibility, and secondary cases of infection.

In addition, detailed examination of amino acid sequences of Brazilian DENV-2 strains isolated in 1998 and 2008 showed 6 aa substitutions in the envelope gene: V129I, L131Q, I170T, E203D, M340T, and I380V. Our results support the notion that aa positions at 129 and 131 in the envelope gene are critical genetic markers for phylogenetic classification of DENV-2 (*7**–**9*).

Notably, residue 131 in the envelope gene is located within a pH-dependent hinge region at the interface between domains I and II of the envelope protein. Mutations at this region may affect the pH threshold of fusion and the process of conformational changes (*10*).

Our results suggest the circulation of genetically different DENV-2 in Brazil and that these viruses may have a role in severity of dengue diseases. These findings can help to further understand the complex dynamic pathogenic profile of dengue viruses and their circulation in dengue-endemic regions.
